# Commissioning and validation of a Monte Carlo algorithm for spine stereotactic radiosurgery

**DOI:** 10.1002/acm2.14092

**Published:** 2023-07-11

**Authors:** Cory Knill, Raminder Sandhu, Brian Loughery, Lifeng Lin, Robert Halford, Doug Drake, Michael Snyder

**Affiliations:** ^1^ Department of Radiation Oncology Corewell Health William Beaumont University Hospital Royal Oak Michigan USA

**Keywords:** brainlab spine SRS, Monte Carlo, versa HD

## Abstract

**Purpose:**

A 6FFF Monte Carlo (MC) dose calculation algorithm was commissioned for spine stereotactic radiosurgery (SRS). Model generation, validation, and ensuing model tuning are presented.

**Methods:**

The model was generated using in‐air and in‐water commissioning measurements of field sizes between 10 and 400 mm^2^. Commissioning measurements were compared to simulated water tank MC calculations to validate output factors, percent depth doses (PDDs), profile sizes and penumbras. Previously treated Spine SRS patients were re‐optimized with the MC model to achieve clinically acceptable plans. Resulting plans were calculated on the StereoPHAN phantom and subsequently delivered to the microDiamond and SRSMapcheck to verify calculated dose accuracy. Model tuning was performed by adjusting the model's light field offset (LO) distance between physical and radiological positions of the MLCs, to improve field size and StereoPHAN calculation accuracy. Following tuning, plans were generated and delivered to an anthropomorphic 3D‐printed spine phantom featuring realistic bone anatomy, to validate heterogeneity corrections. Finally, plans were validated using polymer gel (VIPAR based formulation) measurements.

**Results:**

Compared to open field measurements, MC calculated output factors and PDDs were within 2%, profile penumbra widths were within 1 mm, and field sizes were within 0.5 mm. Calculated point dose measurements in the StereoPHAN were within 0.26% ± 0.93% and −0.10% ± 1.37% for targets and spinal canals, respectively. Average SRSMapcheck per‐plan pass rates using a 2%/2 mm/10% threshold relative gamma analysis was 99.1% ± 0.89%. Adjusting LOs improved open field and patient‐specific dosimetric agreement. Anthropomorphic phantom measurements were within −1.29% ± 1.00% and 0.27% ± 1.36% of MC calculated for the vertebral body (target) and spinal canal, respectively. VIPAR gel measurements confirmed good dosimetric agreement near the target‐spine junction.

**Conclusion:**

Validation of a MC algorithm for simple fields and complex SRS spine deliveries in homogeneous and heterogeneous phantoms has been performed. The MC algorithm has been released for clinical use.

## 1 INTRODUCTION

Spinal metastases develop in over 33% of cancer patients and have historically been treated in part with radiotherapy consisting of conventional dose fractionations for pain palliation.^1^, ^2^ More recently, stereotactic body radiation therapy (SBRT) has been adopted for the treatment of spinal metastasis, particularly for oligometastasis, due to the benefits of increased survival and longer pain mitigation.^3^, ^4^ With increased survival times, SBRT failures have been found to occur primarily in the bone adjacent to the previous treatment and the epidural space adjacent to the spinal cord.^3^, ^5^, ^6^, ^7^, ^8^, ^9^ To prevent failures in the bone due to marginal misses, the International Spine Research Consortium have published contouring guidelines that divide the vertebrae into subregions, where entire subregions are treated based on disease in a part of the subregion.^10^ Failures in the epidural space may be prevented with expansion of the target volume into the vertebral foramen, however this is limited by the spinal cord tolerance. Thus, a typical spine SBRT target involves one or more subregions of the vertebra that surrounds the dose limiting spinal cord.

Treatment plan generation for spine SBRT has traditionally been a limiting factor in the treatment paradigm due to poor optimization in concave targets surrounding avoidance structures, resulting in suboptimal target coverage. This led the American Association of Physicists in Medicine's Task Group 119 to recommend the use of a simulated spine delivery to push the limits of optimization and delivery abilities during commissioning.^11^ To circumvent the planning optimization difficulty for SBRT spine treatment, Brainlab (Munich, Germany) created a dedicated spine optimization algorithm, that is, built into their Elements Spine SRS v3.0 treatment planning system. The algorithm is tailored for the concave geometry of SBRT spine and has been shown to produce plans with improved conformity and gradient indices while reducing spinal cord dose.^12^, ^13^, ^14^, ^15^, ^16^, ^17^

Although Elements Spine SRS may be able to generate improved clinical dose distributions, the benefit will only be realized for the patient if the dose is accurately delivered in the treatment room. Dosimetric inaccuracies could lead to a gross tumor volume (GTV) underdose, which has been shown to increase recurrences and decrease local control.^18^ Therefore, rigorous validation of the calculation algorithms used in the software, along with the deliverability of the plans on the treatment machine, is necessary.

The Spine SRS Element uses two dose calculation engines: (1) pencil beam calculation (PBC) model that uses lookup tables for dose calculations, (2) Monte Carlo (MC) model based on the X‐ray Voxel Monte Carlo algorithm.^19^, ^20^, ^21^, ^22^, ^23^, ^24^, ^25^ For MC planning, the initial optimization and calculation are performed with PBC to reduce time. After initial optimization, the user has the option to re‐calculate and re‐optimize dose with MC. The beam data collection, commissioning, and validation of the PBC algorithm were previously performed as part of a cranial single‐isocenter multi‐target commissioning.^26^ The previously‐published validation of PBC for simple fields is applicable to the Spine SRS Element commissioning as well. This article will focus on the beam data collection and generation of the MC model, the validation of MC for simple fields, and finally the validation of PBC and MC for more complex spine deliveries.

## 2 METHODS

The MC model was commissioned for the 6MV flattening filter free (6FFF) energy on an Elekta Versa HD (Stockholm, Sweden). Commissioning measurements were performed in both air and water. In‐air measurements included percent depth doses (PDDs), longitudinal and transverse profiles, and collimator scatter factors. In‐water measurements included PDDs, longitudinal and transverse profiles, output factors, and absolute dose calibration. Measurements were performed in a 3DS tank using a 125c reference detector (Sun Nuclear Corporation, Melbourne, FL, USA). A piece of lead was removed from the head of the linac, and the reference detector was placed above the jaw as to not perturb the field. Output factors were measured using both microDiamond model 60019 (PTW, Freiburg, DE, USA) and Edge (Sun Nuclear Corporation, Melbourne, FL, USA) detectors. In‐air measurements were performed with a 2 mm brass buildup cap.

Commissioning measurements included field sizes down to 10 mm x 10 mm, which necessitated the use of correction factors for small field measurements. Small field output factors were measured using a daisy‐chain method where the small field detectors (microDiamond and Edge) were cross calibrated with a 125c chamber at a 30 mm x 30 mm field size. For smaller field sizes, correction factors were applied using the Alfonso formalism with the intermediate daisy‐chain field size selected as 30 mm x 30 mm.^27^ The equation used to calculate output factors for a given clinical field size ($\Omega_{Q_{\mathrm{clin}},Q_{100\mathrm{mm}\;x\;100\mathrm{mm}}}$) was:

(1) $$\begin{array}{ccc} \Omega_{Q_{\mathrm{clin}},Q_{100\mathrm{mm}\;x\;100\mathrm{mm}}} & = & \;{\left[\frac{M_{Q_{\mathrm{clin}}}^{f_{\mathrm{clin}}}}{M_{Q_{30\mathrm{mm}\;x\;30\mathrm{mm}}}^{f_{30\mathrm{mm}\;x\;30\mathrm{mm}}}}\cdot \frac{k_{Q_{\mathrm{clin}}}^{f_{\mathrm{clin}}}}{k_{Q_{30\mathrm{mm}\;x\;30\mathrm{mm}}}^{f_{30\mathrm{mm}\;x\;30\mathrm{mm}}}}\right]}_{\det}\\ & & \cdot {\left[\frac{M_{Q_{30\mathrm{mm}\;x\;30\mathrm{mm}}}^{f_{30\mathrm{mm}\;x\;30\mathrm{mm}}}}{M_{Q_{100\mathrm{mm}\;x\;100\mathrm{mm}}}^{f_{100\mathrm{mm}\;x\;100\mathrm{mm}}}}\cdot \frac{k_{Q_{30\mathrm{mm}\;x\;30\mathrm{mm}}}^{f_{30\mathrm{mm}\;x\;30\mathrm{mm}}}}{k_{Q_{100\mathrm{mm}\;x\;100\mathrm{mm}}}^{f_{100\mathrm{mm}\;x\;100\mathrm{mm}}}}\right]}_{\mathrm{IC}} \end{array}$$

where “*det*” were the detector measurements (*M*) and “*IC”* were the ion chamber measurements. Two sets of correction factors (*k*) for the Edge and microDiamond were taken from TRS‐483 and Casar.^27^, ^28^, ^29^ The final measured output factors were an average of four data points generated by applying the two different correction factors to the two detector measurements.

The commissioning data were sent to Brainlab for model generation. The subsequent model was imported into the Elements treatment planning system for validation. The dosimetric validation of the model followed MPPG5.a guidelines, beginning with simple commissioning fields on a water phantom before progressing to clinical plans on largely homogenous phantoms and finally end‐to‐end (E2E) tests on an anthropomorphic phantom.^30^

To validate the MC model for simple commissioning fields, a large (30 cm^3^) square phantom with density of 1 g/cm^3^ was created in Elements to simulate a water tank. Various static commissioning fields were calculated on the water phantom and compared to measurements. MC calculations were performed using 1% uncertainty, 1 mm dose grid, dose‐to‐medium setting. The accuracy of the calculated PDDs, profiles, output factors, and absolute doses were validated.

Unlike a non‐stochastic PBC algorithm, which will return the same answer for identical calculations, the stochastic MC algorithm will return different answers that vary based on the user‐selected allowable uncertainty in the calculation. This MC uncertainty is easily visible in the PDDs and profiles. However, if a single calculation or voxel is used during output factor or absolute dose validation, the uncertainty may disguise a commissioning error. To mitigate this uncertainty in point dose calculations, each calculation was performed three times using 1%/1 mm parameters, re‐seeding the algorithm in between, and the results were averaged. The algorithm was re‐seeded by recalculating dose using dose‐to‐water 5%/5 mm parameters in between the higher precision dose‐to‐medium calculations. Further discussion of the necessity of re‐seeding the algorithm and the effect of the statistical uncertainty parameter is presented in the discussion section.

In addition to averaging multiple MC calculations, the reference dose for detector measurements were calculated as the mean to a volumetric structure, rather than an individual voxel dose. Provided the detector is sufficiently large to span more than one voxel, calculating dose as a mean of the detector structure will reduce the overall uncertainty in the reference dose. This was done for point dose measurements in the phantom calculations that are described in the subsequent sections.

Commissioning field validation was followed by validation of clinical deliveries in homogeneous phantoms. Nine previously‐treated spine SBRT patients, planned in the Pinnacle (Philips, Amsterdam, the Netherlands) treatment planning system, were selected as a subset for clinical plan evaluation. Patients were selected to represent a comprehensive sampling of both location of the target along the length of the spine as well as the extent of target throughout the vertebrae. The patient's DICOM CT and structure sets were anonymized, imported into the Spine SRS Elements, and re‐planned.

Elements is a template‐based planning system that uses two sets of templates for each plan: prescription and geometry. These templates are site‐specific and for this work can only be applied to spine treatments. The prescription templates dictate the target and OAR constraints, while the geometry templates dictate the beam arrangements. All plans were allowed to have up to six coplanar (couch angle of 0°) partial 180° arcs. The number of arcs was automatically determined by the optimizer's arc‐splitting algorithm, whereby if a target wraps around the spinal canal, creating a cavity, the optimizer will split the PTV into subsections. Each subsection will be treated by one of the overlapping arcs, thereby eliminating the concavity from the optimization and delivery, with the goal of producing sharper dose falloff and organ at risk (OAR) sparing.

The prescriptions and OAR objectives for plan optimization were selected to match the objectives from the original clinical plans. OAR objectives were mainly derived from TG‐101 tolerances.^31^ Target optimization was performed with the goal of achieving Paddick Inverse Conformity Indices (ICIs) of less than 1.2 and Paddick Gradient Indices of less than 4.0.^15^, ^16^ Prescriptions ranged from 16 to 27.5 Gy (simultaneous integrated boosts up to 35 Gy) delivered in 1−5 fractions. Elements‐optimized plans were normalized to achieve at least the same minimum coverage to 95% of the PTV and SIB as the clinical plans.

The normalized Elements plans were compared to the clinical plans using maximum dose to the spinal canal, ICIs, and GIs. Plan MU and delivery time were not compared as most of the original plans were treated using a 6X flattened beam while the Elements plans were generated using a 6FFF beam. Due to the different nature in the beam delivery, the goal of the evaluation was not to directly compare the Pinnacle and Elements optimizers, rather it was to ensure the Elements‐generated plans would be deemed clinically acceptable.

All plans were initially optimized with the PBC algorithm, followed by a re‐calculation with MC without further optimization to evaluate the differences in dose calculated by the beam models. All plans were subsequently re‐optimized using the MC algorithm and exported for dosimetric validation on the Versa HD.

To evaluate dosimetric accuracy, the clinical plans were recalculated on the StereoPHAN (Sun Nuclear, Melbourne, FL, USA) phantom in Elements using the MC model. The MC dose was calculated on a per‐field basis using a 1% uncertainty, 1 mm dose grid, dose‐to‐medium setting. Exported clinical plans were delivered to the SRSMapcheck (Sun Nuclear Corporation, Melbourne, FL, USA) and microDiamond in the StereoPHAN phantom and the results were compared to Elements predicted dose. The orientation of the SRSMapcheck was selected such that the detector plane passed through isocenter and the center of the spinal canal. This allowed measurement of the important dose falloff near the target‐spinal cord junction. Per TG‐157 recommendations, the MC predicted dose was compared to the SRSMapcheck measurements using a 2%/2 mm/10% relative gamma analysis.^32^

microDiamond point dose measurements were performed at planned isocenter and the center of the spinal canal. For spinal canal measurements, the phantom had to be shifted to place the microDiamond at the center of the spinal canal. An additional Elements StereoPHAN calculation, corresponding to the shifted phantom location, was performed for comparison.

After initial StereoPHAN measurements, model tuning was performed by varying the light field offset (LO) distance between the physical and radiological positions of the collimating jaws and MLC in the model, with goal of improving the accuracy of the open field sizes and StereoPHAN calculations. LO adjustments were made in the Brainlab Physics Administration software version 6.0. After each LO adjustment, the dose to the StereoPHAN was recalculated in Elements and the results were compared to the previous measurements. In addition, the MC‐predicted field sizes and output factors were recalculated for each LO adjustment.

LO adjustments were done in an iterative manner to get an optimal value. Once an optimal LO value was identified, the model was returned to Brainlab for further optimization. This additional optimization involved manually tuning the primary source sizes to improve the penumbras of small fields, while maintaining the same LO. The re‐optimized model was imported into the treatment planning system and re‐evaluated with regards to predicted field sizes, output factors, and clinical dose deliveries on the StereoPHAN.

After dosimetric validation in homogeneous phantoms was complete, the dose was verified in a heterogeneous spine phantom, produced by RTsafe P.C. (Athens, Greece), that consisted of vertebrae‐like structures that were suspended within a water‐filled body cavity. The phantom had two holes inside the vertebral body and spinal canal that allowed for microDiamond point dose measurements. The holes were replaced with PMMA plugs for CT acquisition and dose calculations. A CT image set using 1 mm slice thickness was acquired of the phantom, imported into Elements, and a PTV structure was created on phantom to cover the microDiamond in the vertebral body, while avoiding the spinal canal. A plan was generated using an initial PBC optimization, followed by recalculation with MC and re‐optimization.

The plan was exported to the record and verifies system for delivery. Positioning of the phantom was performed using ExacTrac (Brainlab, Munich, Germany) to replicate clinical practice. The plan was delivered to the phantom while measuring dose with the microDiamond that was subsequently compared to the dose predicted by the MC calculation. The microDiamond was calibrated in the StereoPHAN with the reference dose calculated using the MC model with a phantom electron density of 1.20 g/cm^3^ relative to water, which was selected to match the approximate density of the PMMA plugs that were placed within the spinal canal. The same density overrides were applied to the chamber inserts in the heterogeneous phantom and the dose was calculated as dose‐to‐medium.

Due to the design of the phantom, dose measurement in the vertebral body placed the detector in the axial plane. This made the measurement susceptible to the angular dependency of the microDiamond. To correct for this dependency, the angular dependency of the microDiamond was measured and used to measurements in the vertebral body. Angular dependency correction factors were measured by placing the microDiamond in the StereoPHAN cube insert (without the StereoPHAN) at isocenter with the detector in the axial (gantry rotation) plane. The microDiamond was irradiated with 100 MU from a 50 mm x 50 mm 6FFF field for all gantry angles in 10° increments. The measurement was repeated with the microDiamond located in the sagittal plane along the length of the couch. The axial plane results were normalized with the sagittal measurements to correct for any variations in the machine output for different gantry angles. The corrected measurements were then normalized to gantry angle zero to determine the angular dependency of the microDiamond.

Two types of angular corrections were generated using the measured microDiamond angular dependency. A simple correction was derived by averaging all angular dependency measurements to generate a global correction factor that could be used for any axial measurements. In addition, a more advanced correction was generated by writing software that queried the DICOM RT plan file to obtain MU delivered per gantry angle, which was multiplied by the gantry‐angle specific microDiamond dependency factors to obtain a correction factor for individual deliveries.

In addition to the angular corrections, the RTsafe phantom irradiation introduced the need for couch‐attenuation corrections as the phantom was placed on the body of the 4D tabletop (unlike the StereoPHAN, which was hung off the end of the table). Elements has pre‐configured couch models that can be positioned below the patient on CT import. To validate the accuracy of the models, couch attenuation measurements were performed and compared to the corresponding calculations in Elements. Attenuation was measured by placing the ArcCHECK (Sun Nuclear Corporation, Melbourne, FL, USA) directly on the Elekta Hexapod tabletop (no stand) and measuring dose at various gantry angles for a 6FFF 100 mm x 100 mm field using an A12 chamber (Standard Imaging, Middleton, WI, USA) located in the center of the ArcCHECK middle insert. Measurements at each gantry angle were normalized to gantry zero to obtain table transmission, which were subsequently compared to MC calculations.

Final dose verification was performed using an insert filled with polymer gel (VIPAR base forumation), that was inserted into a homogeneous region of the RTsafe spine phantom. Previously treated clinical target and spinal cord structures were copied to phantom and a plan was generated to obtain 95% coverage of prescription dose to the target, while minimizing spinal canal dose. The plan was delivered to the phantom using on‐board cone‐beam CT for alignment. ExacTrac was not used for positioning due to a lack fo heterogenous tissue in the homogeneous phantom. An MR acquisition of the phantom was performed the following day and sent to RTsafe for dosimetric analysis.

## 3 RESULTS

**FIGURE 1:**
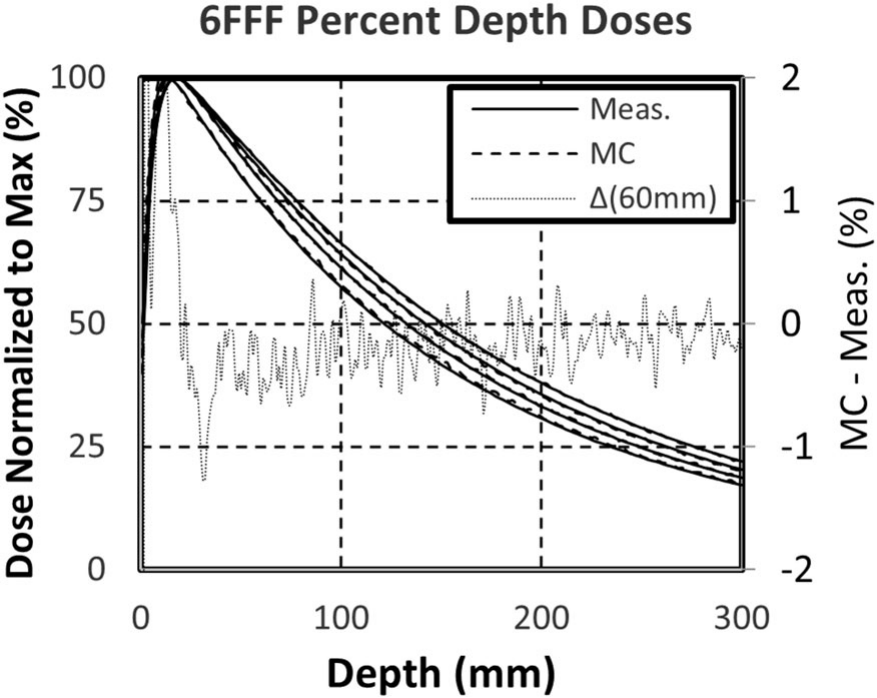
Measured (Meas.) and Monte Carlo (MC) calculated percent depth doses (PDDs) for field sizes of 100, 60, 30 mm, and 10 mm at 900 mm SSD. The difference between the Measured and calculated PDDs for the field size with the largest deviation past dmax (60 mm) is plotted as Δ(60 mm).

**FIGURE 2:**
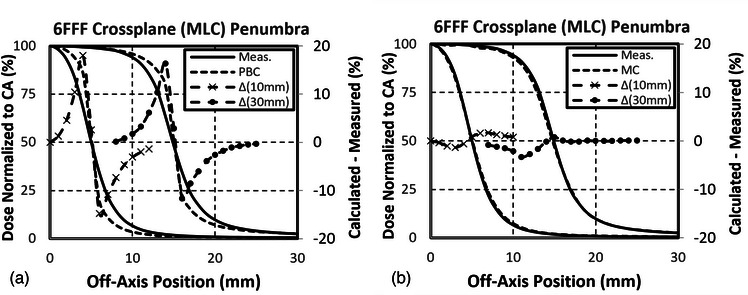
Comparison of PBC (a) and MC (b) calculated crossplane profiles with commissioning measurements for 30 mm × 30 and 10 mm × 10 mm fields at 900 mm SSD and 100 mm depth.

The 6FFF MC‐calculated PDDs were within 2% of measured past the depth of maximum dose, with most points falling within 1% (Figure 1). Calculated profiles were within 2% of measured in the middle of the field (80% of full‐width half‐maximum [FWHM] of the profiles). The MC‐predicted penumbras were more accurate than PBC‐predicted penumbras, particularly in the crossplane (MLC) direction (Figure 2). The final calculated penumbra widths (80–20%) were within 1 and 2.5 mm of commissioning for MC and PBC, respectively. Increasing the LO parameter reduced the FWHM of the fields. LO adjustments were in part performed to improve the agreement between calculated and measured FWHMs across all field sizes and depths. The final calculated FWHMs were within 0.5 mm of measured after LO adjustments.

Calculated output factors were within 1.5% of measured after LO adjustments for square and irregular fields with jaw and MLC widths between 10 and 60 mm (Table 1). For validation, all output factors were calculated and measured with guard leaves open (two open MLCs behind both collimating jaws) to mimic clinical deliveries. Increasing the LO parameter reduced the calculated field size and subsequently reduced the calculated small field output factors. Re‐optimization of the model, while keeping the LO parameter constant, increased the discrepancy between the commissioning and calculated 10 mm output factor, while reducing the discrepancy for 20 and 30 mm output factors. Finally, the MC‐calculated absolute dose calibration was within 0.25% of measured for a 100 mm × 100 mm field at 900 mm source‐to‐surface distance (SSD) and 100 mm depth.

**TABLE 1:** Calculated Monte Carlo (C) output factors.

6FFF calculated output factors [difference between calculated and measured]							
		Jaw positions					
		10 mm	20 mm	30 mm	40 mm	50 mm	60 mm
MLC positions	10 mm	0.715 [−0.5%]	0.759 [−0.3%]	0.778 [ 0.5%]	0.779 [−0.1%]	0.782 [−0.2%]	0.784 [−0.3%]
	20 mm	0.769 [0.1%]	0.830 [0.0%]	0.854 [1.0%]	0.860 [0.4%]	0.867 [0.5%]	0.871 [0.6%]
	30 mm	0.790 [0.8%]	0.854 [0.6%]	0.868 [−0.3%]	0.888 [0.6%]	0.892 [0.0%]	0.900 [0.3%]
	40 mm	0.791 [−0.2%]	0.865 [0.5%]	0.892 [0.8%]	0.905 [0.7%]	0.911 [0.1%]	0.927 [1.1%]
	50 mm	0.793 [−0.4%]	0.874 [0.7%]	0.898 [0.4%]	0.912 [0.2%]	0.929 [0.7%]	0.938 [0.6%]
	60 mm	0.802 [0.3%]	0.877 [0.5%]	0.912 [1.2%]	0.923 [0.3%]	0.941 [0.7%]	0.947 [0.3%]

*Note*: Comparison to measured (*M*) output factors are shown in the brackets as [(C—M)/M × 100%].

**FIGURE 3:**
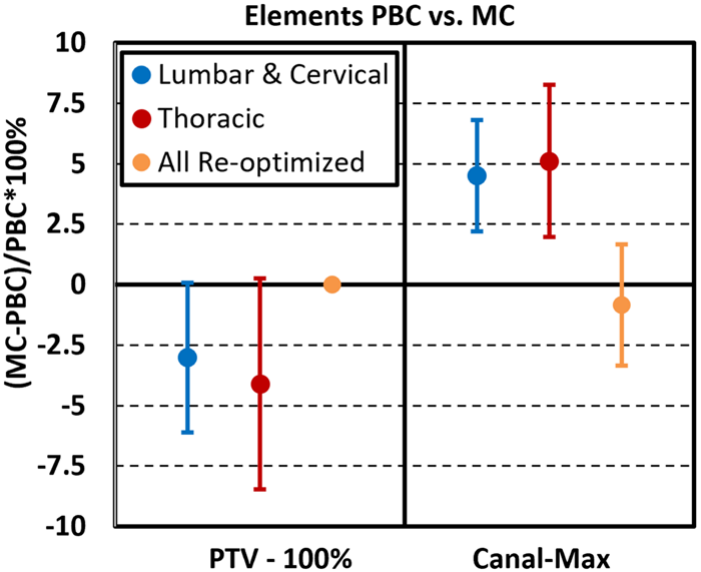
Comparing dosimetric differences in PTV coverage and max canal dose between PBC‐optimized plans that were recalculated with MC. Cases were separated for targets in the heterogeneous thoracic region and more homogeneous lumbar and cervical regions. All cases were re‐optimized with MC, re‐normalized to match PBC PTV coverage, and the canal dose between the two algorithms was compared.

Figure 3 shows the difference in percent of PTV covered by prescription dose (PTV‐100%) and maximum spinal canal dose (Canal‐max) between PBC plans that were re‐calculated with MC without further optimization. Figure 3 also shows that after MC re‐optimization (orange) and normalization to the same PTV coverage, the average MC calculated spinal canal doses were within 1% of PBC.

**FIGURE 4:**
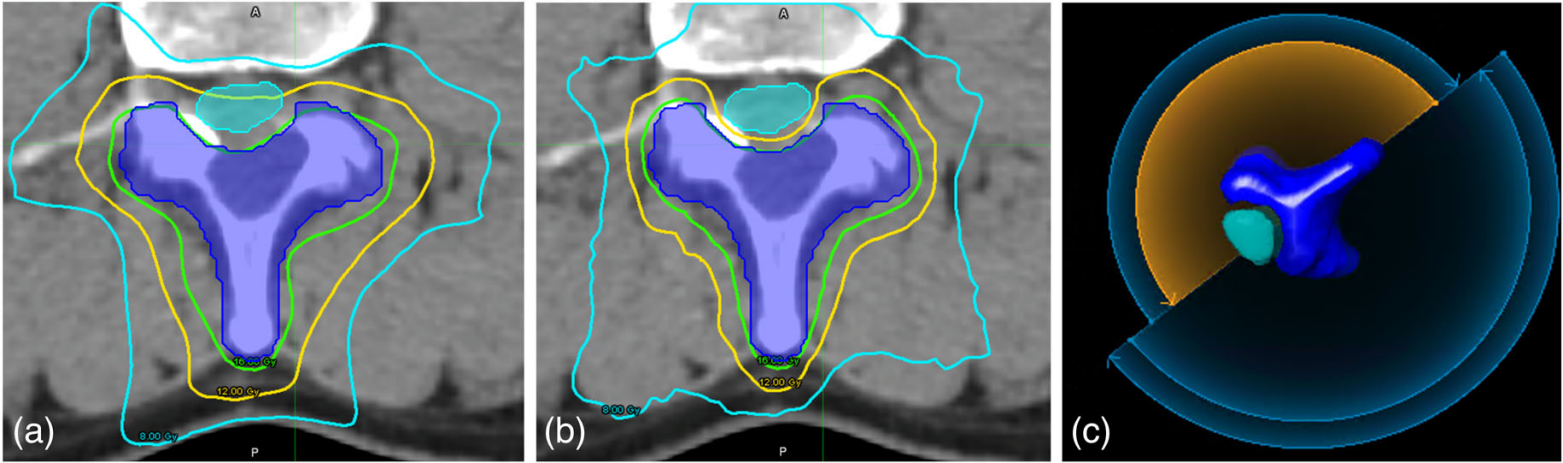
Comparison of (a) Pinnacle and (b) Elements isodose lines for a concave target around the spinal canal. (c) Arc splitting in the Elements optimization.

Elements MC re‐optimized plans reduced the Canal‐max by 3.59 ± 2.13 Gy, while maintaining the same PTV coverage as Pinnacle. Elements re‐optimized plans improved the average ICI by 2.30% ± 9.12% and the average GI by 16.98% ± 15.71%. Based on this analysis, the Elements plans were deemed clinically acceptable for treatment. Figure 4 shows the arc‐splitting function in the Brainlab optimization that splits a concave target into two separate targets, thereby minimizing the concavity treated for each field. This allows sharper falloff of the isodose lines in the target‐spinal canal junction.

**FIGURE 5:**
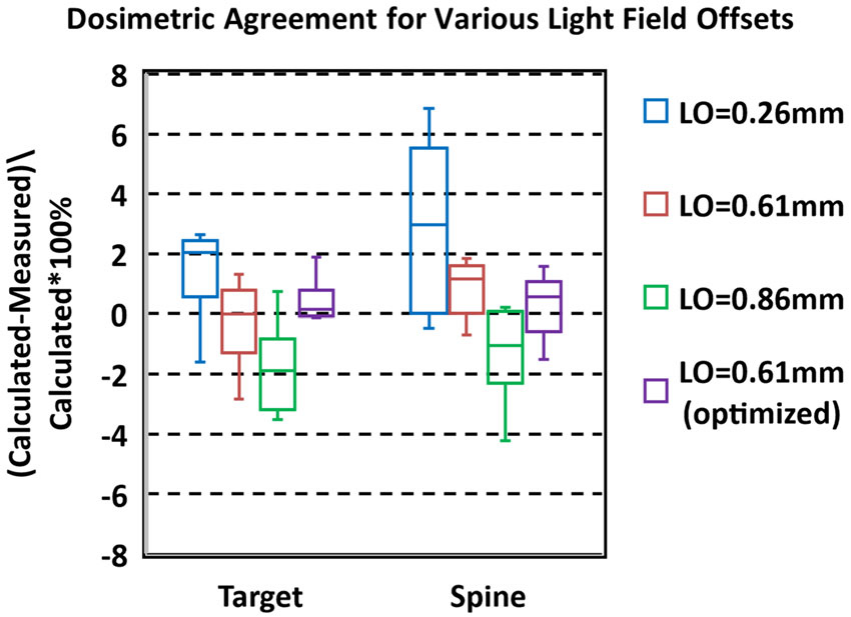
Difference between measured and calculated dose for clinical cases in a homogeneous StereoPHAN phantom for various light field offset (LO) values. LO = 0.61 mm (optimized) used an adjusted LO of 0.61 mm and had additional model tuning to improve accuracy.

Increasing the LO parameter from the initial 0.26 to 0.61 mm improved target and spinal canal dose for clinical cases (Figure 5). Additional optimization of the Monte Carlo source models by Brainlab, with the optimal LO of 0.61 mm shown in Figure 5, further improved the dosimetric agreement of the clinical cases. An alternative LO adjustment technique could be performed by directly minimizing the FWHM error between measured and calculated profiles for various field sizes and depths. However, it is recommended to validate the impact of these adjustments through measurements of target and OAR doses in clinical plans, as any dosimetric discrepancy for these plans will directly impact patient treatments.

On average, microDiamond measured point doses in the StereoPHAN were within 0.26% ± 0.93% and −0.10% ± 1.37% for targets and OARs, respectively. Absolute differences were 0.63% ± 0.70% and 0.84% ± 0.60% for targets and OARs, respectively. Average per‐plan pass rates using a 2%/2 mm/10% threshold relative gamma analysis was 99.1% ± 0.89%.

microDiamond measurements in the heterogeneous RTsafe phantom were within −1.29% ± 1.0% and 0.27% ± 1.36% of MC calculated for the vertebral body (target) and spinal canal (OAR), respectively. When compared to PBC calculated dose, the magnitude of those differences increased to −2.62% and 6.05% for the vertebral body (target) and spinal canal (OAR), respectively. The microDiamond angular correction factor for the target measurements with the detector in the axial plane was 2.50% for the simple correction, calculated by averaging corrections over all gantry angles. The more advanced complex corrections, calculated using the DICOM RT plan file, were 2.57%.

**FIGURE 6:**
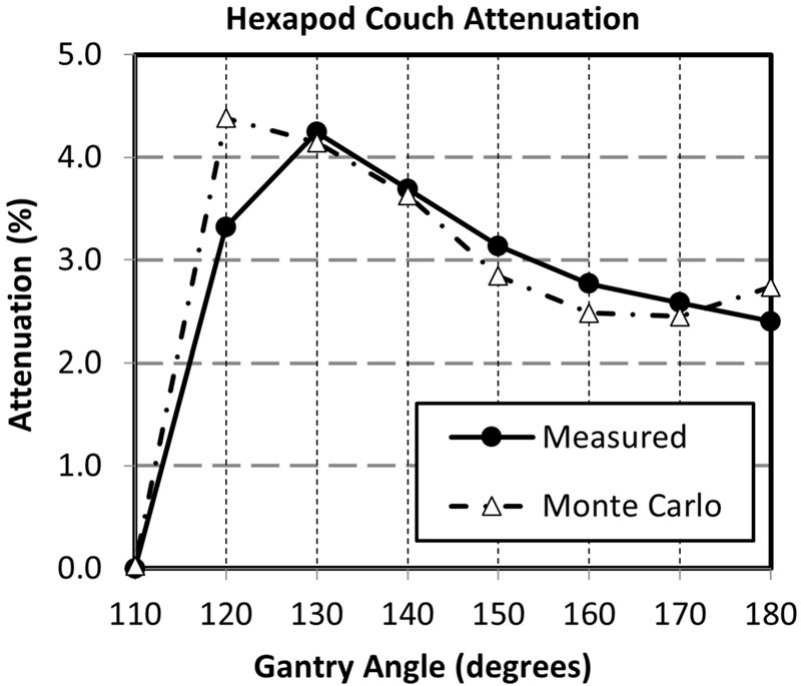
Comparing the measured versus Monte Carlo calculated couch attenuation of the main body of the Hexapod couch for 6FFF.

The couch‐attenuation calculations and measurements are shown in Figure 6. Most of the calculated values were within 0.4% of the measured values. The largest discrepancy of 1.1% occurred when the beam passed through the edge of the couch at gantry angle 110°. For a 180° arc, with uniform dose delivery, the average expected error in calculated dose due to couch‐modeling is 0.03%. The measured attenuation of 2.4% at gantry angle of 180° matched the attenuation values stated for a 6X beam in Elekta's Hexapod manual.

**FIGURE 7:**
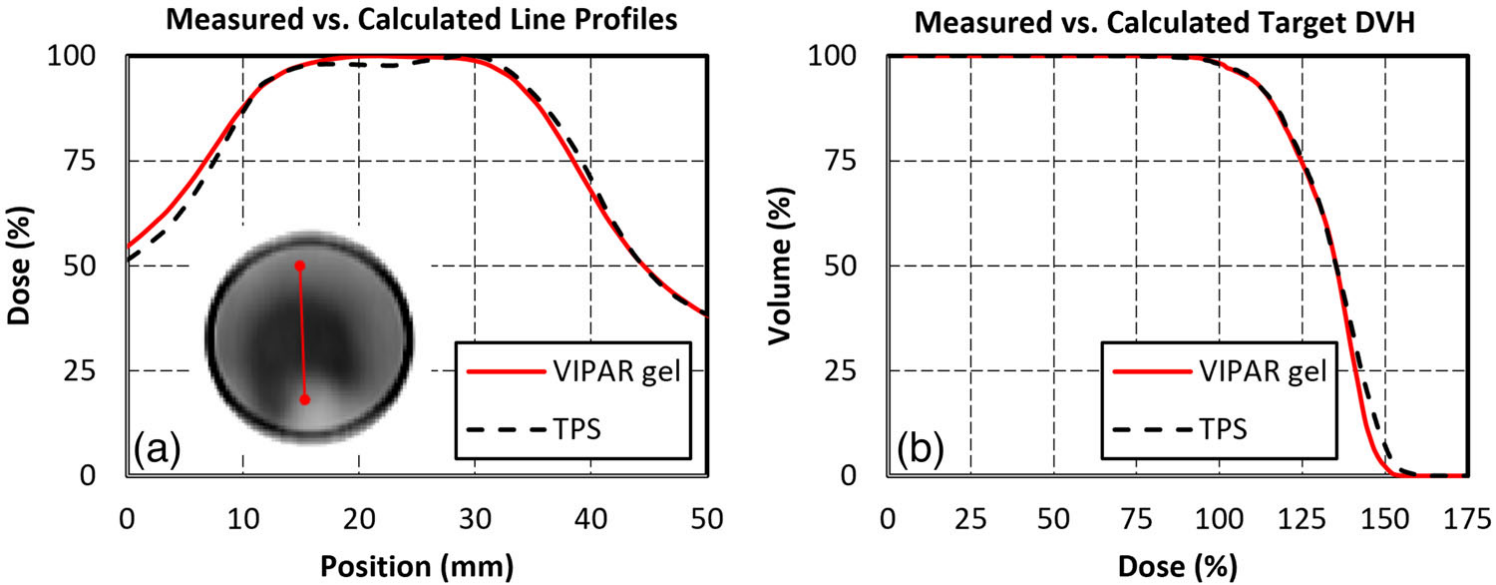
RTsafe polymer gel dosimetric results. (a) Line profile showing the Monte Carlo calculated and VIPAR gel measured dose in the target‐spinal canal junction. (b) Calculated (TPS) versus measured (RTsafe) target DVHs.

A line profile of the VIPAR gel results showing the target–spinal canal junction are shown in Figure 7a. The mean dose to the target measured by the VIPAR gel was 0.80% higher than the Monte Carlo predicted dose. The target DVH predicted by Monte Carlo and VIPAR gel are overlayed in Figure 7b.

## 4 DISCUSSION

The accuracy of the Elements MC algorithm was successfully validated for simple fields and clinical spine SRS plans in both homogeneous and heterogeneous phantoms. The validation of the MC algorithm involved unique challenges due to the stochastic nature of the calculation, along with the assumptions regarding material properties within the phantom CTs. Unlike PBC, which uses lookup tables to calculate dose, the Elements MC algorithm calculates dose using tissue‐specific dose deposition parameters (electron stopping powers, Compton scattering cross sections, mass densities, etc.). These parameters are derived from the CTs Hounsfield Unit values, with the presumption that the CT contains physiological tissue of a patient. However, this is not the case for the phantoms that are typically used in dose verification, which contain non‐biological tissue such as polymethyl methacrylate (PMMA).

To overcome this assumption, it is recommended that TPR or PDD data are acquired within the phantom material and the corresponding measurement is calculated in the planning system using phantom overrides of various densities. The phantom density which produces the optimal fidelity between measurements and calculations should be used for subsequent commissioning measurements. Prior to investigating phantom densities, it is important to first validate the accuracy of the MC‐calculated PDDs in water. This will ensure that the density selection of the phantom does not disguise fundamental errors in the PDDs of the MC model.

In addition to the appropriate selection of phantom densities, the uncertainty and grid size used in commissioning must be appropriately selected to yield sufficiently accurate calculations in an efficient amount of time. The accuracy of each calculation is dictated by the user‐selected percent uncertainty parameter. In MC, the uncertainty of any given voxel is defined as $1/\sqrt{N}$, where *N* is the number of events that are counted in that voxel. During an MC calculation, the algorithm will continue to calculate more events until the uncertainty in the maximum dose voxel equals the user‐selected parameter. The resulting uncertainty in non‐max‐dose voxels may be higher due to less events being counted in those voxels during calculation.

Lowering the uncertainty parameter will improve accuracy; however, it increases calculation time by a factor of *N* ^2^. The additional time constraint can be particularly troublesome if the system is being simultaneously used clinically as calculations will need to be halted to plan cases. One option is to run these calculations with a low uncertainty parameter over nights and weekends to increase precision. However, this often requires remote connectivity to start the next calculation and can greatly slow down commissioning and validation progress due to calculation times. Another option is to run the calculations multiple times with a higher uncertainty and average the results. The potential advantage of running multiple calculations with higher uncertainty is two‐fold. (1) If a calculation needs to be halted so that the planning system can be used for a clinical case, less data will be lost. (2) Running calculations with higher uncertainty is particularly useful during initial investigations as it can be used to quickly identify poor results that may be caused by deficiencies in the beam model or incorrect calculation setups (e.g., incorrect SSDs). In addition to manipulating the uncertainty parameter, the calculation time can be reduced by increasing the calculation grid size. On average, adjusting the parameters from 1%/1 mm to 2%/2 mm resulted in a 94% reduction in calculation time. For geometries requiring the longest calculation times, large fields on large phantoms, this adjustment reduced a 45‐min calculation to approximately 3 min.

When running multiple MC calculations, it is important to note that the initial particle histories used in the calculation are dependent on the internal beam IDs. One issue with running consecutive MC calculations with no change in calculation parameters is that the MC algorithm will use the same particle histories and produce the same calculation result. Therefore, it is necessary to re‐seed the algorithm in between calculations, which can be done by changing the calculation type to dose‐to‐water with a larger grid size and higher calculation uncertainty (to increase calculation speed), running a calculation, and then returning the calculation to dose‐to‐medium with the more accurate calculation settings. Alternatively, the algorithm can be re‐seeded by restarting the calculation module without any dose calculated and re‐computing the dose.

Furthermore, the stochastic nature of the MC calculation may affect the overall pass rates of the SRSMapcheck gamma calculations. Graves et al. reported that noise in the reference dose of a gamma calculation, like that from the uncertainty in a MC calculation, may increase the pass rates of the gamma calculation.^33^ Pass rates will continue to improve as the magnitude of the noise increases, which would correspond to an increase in uncertainty in the MC calculation. For a further discussion of the nuances of MC implemented the readers are referred to TG‐105 and TG‐157.^34^, ^35^

As shown in Figure 3, the initial PBC calculated dose in both the heterogeneous thoracic region and more homogeneous lumbar and cervical regions both deviated from the MC re‐calculated dose. However, after MC re‐optimization, the resulting PTV coverage and OAR sparing was like what was achieved in the initial PBC calculation. This suggests that initial optimizations using PBC can provide a fast method for exploring realizable optimization objectives that could be achieved with subsequent MC re‐calculation and re‐optimization.

While the use of PBC in initial optimization allows for the fast exploration for a realizable optimization solution, it is important to note that PBC reports dose‐to‐water, while MC reports dose‐to‐medium. AAPM Task Group 329 (TG‐329) addresses the differences in dose reporting and estimates that the MC reported dose‐to‐medium will be approximately 1% lower than the PBC reported dose‐to‐water in soft tissue.^36^ The magnitude of this discrepancy in cortical bone can increase to over 10%.^37^

To resolve this discrepancy, TG‐329 recommends reducing the PBC absolute dose calibration by 1% to simulate dose‐to‐medium calculations. This will reduce the discrepancy between the two dose calculation algorithms due to dose reporting, which may reduce the frequency at which additional MC optimization is needed. However, within the Brainlab Elements environment, the PBC algorithm may also be used for dose calculations for cranial SRS, where MC calculations are less common and historical dose‐prescriptions from Elekta's Gamma Knife planning system (Gamma Plan) were reported as dose‐to‐water. Due to the multi‐anatomy use of the PBC algorithm, along with the discrepancy in bone between the dose reporting methods, the decision was made to not adjust the PBC calibration to dose‐to‐medium, rather the MC calculation will be used for all spine SRS cases. This will ensure consistent dose reporting and will also take advantage of the improved heterogeneity corrections and penumbra modeling in MC compared with PBC. Furthermore, it is recommended to discuss these discrepancies with the prescribing physician to maintain full transparency in dose reporting.

The microDiamond measurements in the vertebral body of the heterogeneous phantom required correction factors >2% due to the location of the detector in the axial plane, which caused direct radiation delivery through the stem of the detector. Fortunately, simply averaging the overall detector angular dependency measured with 10° gantry increments resulted in a correction factor that was within 0.07% of a more complicated correction factor calculated from the DICOM files for the plan. It is recommended for future phantom development that both the detector in the vertebral body and spinal canal be located along the axis of the spine, to eliminate the need for angular correction factors. When the detectors are not in use, plugs consisting of the same material surrounding the cavity should be used to fill the void.

The dosimetric measurements in the heterogeneous phantom allowed for both validation of heterogeneous calculations and a CT‐to‐delivery end‐to‐end test as the ExacTrac was used to position the phantom. The measured dose matched more closely with the MC calculation compared to the PBC calculation in the heterogeneous phantom, particularly in the spinal canal. This further highlights the need for the more accurate MC calculation algorithm in spine SRS planning. Overall, the improvement was likely a combination of improved heterogeneity corrections in the MC calculations as well as improved beam modeling, particularly in the crossplane penumbra modeling of the leaf tip (Figure 2).

Although the accuracy of the MC dose calculation was verified in the presence of bone heterogeneities, a spine SRS procedure may occur in the thoracic region where significant air is present or after reconstructive surgery where high‐density metal is implanted in the patient. Previous publications have validated the accuracy of the MC algorithm's heterogeneity corrections for air and bone in phantom geometries.^38^, ^39^ However, future anthropomorphic phantom testing could potentially include metal hardware and lung, to validate dose calculation accuracy of clinical plans in the presence of these heterogeneities.

In addition to the good agreement of target and spinal canal dose in the heterogeneous phantom, the VIPAR gel measurements in the target‐spinal canal junction matched closely to the dose calculated by the Monte Carlo algorithm. The measured mean dose to the PTV was slightly higher than the Monte Carlo calculation; however, the less than 1% difference was well within clinical tolerances. The measured and calculated dose profiles exhibited a sub‐millimeter shift. However, these deviations fell within the specified geometric accuracy of 1 mm and dosimetric accuracy of 3%−5% for the gel analysis procedure outlined by RTsafe.

The Elements site‐specific spine SRS optimizer was able to produce plans with lower spinal canal dose and similar target coverage to Pinnacle. Deliverability of these plans was subsequently validated for dosimetric accuracy via patient specific quality assurance. Given the plan quality and dosimetric accuracy, it was determined that the Elements‐generated plans utilizing the MC calculation were acceptable for clinical implementation.

## 5 CONCLUSION

A 6FFF Monte Carlo beam model for a Versa HD was commissioned in Brainlab's Elements treatment planning system. Initial model validation was performed by calculating simple fields in a homogeneous water phantom. Subsequent model validation was performed by optimizing patient plans in the Spine SRS Element and validating dose on the homogeneous StereoPHAN phantom. The model's Light Field Offset parameter was used to optimize the model's calculation accuracy. Finally, MC‐calculated dose delivery was validated using an end‐to‐end procedure on a heterogeneous spine phantom. The dosimetric accuracy of the MC algorithm has been found to be within acceptable clinical limits and has been released for clinical use.

## AUTHOR CONTRIBUTIONS

All authors contributed equally to this work.

## CONFLICT OF INTEREST STATEMENT

This work was supported by a Clinical Cooperation Agreement with BrainLab AG, Munich, Germany.
